# Impact of postpartum anxiety and depression on child’s mental development from two peri-urban communities of Karachi, Pakistan: a quasi-experimental study

**DOI:** 10.1186/1471-244X-13-274

**Published:** 2013-10-22

**Authors:** Niloufer Sultan Ali, Sadia Mahmud, Asia Khan, Badar Sabir Ali

**Affiliations:** 1 Department of Family Medicine, Aga Khan University, Stadium Road, P.O. Box 3500, Karachi 74800, Pakistan; 2 Department of Medicine, Aga Khan University, Stadium Road, P.O. Box 3500, Karachi 74800, Pakistan; 3 Department of Paediatrics and Child Health, Aga Khan University, Stadium Road, P.O. Box 3500, Karachi 74800, Pakistan

**Keywords:** Postpartum anxiety and depression, Children, Mental development

## Abstract

**Background:**

Postpartum anxiety and depression has detrimental effects on the overall mental development of children. This study aims to assess the impact of postpartum anxiety and depression on children’s mental development on all sub-scales in a Pakistani population.

**Methods:**

A quasi-experimental study was conducted in two peri-urban communities of Karachi, a mega city of Pakistan, to assess the impact of postpartum anxiety and depression on children’s growth and mental development. A total of 420 women were enrolled, who had given consent out of 651 pregnant women identified, during February 2004 to December 2005. Data for socio-demographic, home environment and family relationship variables were collected between 36 weeks of pregnancy and within 10 days of childbirth. Mother’s levels of anxiety and depression were assessed at 1, 2, 6, 12, 18, 24, and 30 months of childbirth. An indigenous, validated screening instrument- Aga Khan University Anxiety and Depression scale was used and diagnostic confirmation was done through a psychologist’s interview, based on DSM IV criteria. Children’s growth and development was monitored in the same sequence using an Early Childhood Development tool that consists of five subscales; socio emotional, language, cognitive, gross motor and fine motor development. Physical growth was monitored by measuring height and weight of the child. Data was analyzed using SAS 9.2. Multivariable Generalized Estimating Equations (GEE) logistic regression was conducted to identify association of postpartum anxiety and depression with each early childhood development indicator, adjusting for parental and child factors.

**Results:**

A significant association of postpartum anxiety and depression with delayed development on all five subscales of children’s mental development was found in our study. Interestingly, our study found that higher maternal age had adverse effects on child’s emotional whereas positive impact on child’s cognitive development. Children’s stunting had an adverse impact on all five subscales of children’s development. Male children were at higher risk for delayed language and gross motor development relative to female children.

**Conclusions:**

Our study found that postpartum anxiety and depression is associated with adverse outcomes regarding children’s mental development on all sub-scales. The impact was accentuated by low family income or child’s increasing age.

## Background

Postpartum depression (PPD) is a major public health problem. It affects up to 15% of mothers in Western societies [1]. However, a review conducted on 143 studies from 40 countries shows that the prevalence of PPD varies from 0.5 – 60% depending on the definitions used and influence of various cultural factors [2]. This wide range might be also due to differences in socio-economic environments e.g. poverty and levels of social support and biological vulnerability factors. Studies conducted in rural sub-district and in urban tertiary care settings of Pakistan have reported a high prevalence of PPD ranging from 24% -56% in Pakistani women [3-8]. Over the past 15 years, there has been growing literature which has shown associations between PPD and adverse child outcome. The consequences of postpartum depression are not restricted to infancy, but can also extend into toddlerhood, school age and even adulthood. Infants of depressed mothers show impaired maternal-child interactions [9], lower cognitive development, more behavioral problems [10], and a higher risk of psychiatric disorders during adolescent years than those of non-depressed mothers [11].

Infancy is a vulnerable period, as at this stage they are dependent on their mothers/caregivers for primary interactions. Black MM. et al. has reported that infants with maternal depressive symptoms had acquired fewer cognitive, motor and orientation/engagement skills than infants whose mothers were non-depressed [12]. Cornish A. M. et al. has found an association of chronic maternal depression lasting throughout the first one year with lower infant cognitive and psychomotor development [13]. A prospective longitudinal study conducted by Sutter-Dalley A.L. et al. has reported that children of mothers with early postnatal depressive symptoms were significantly more likely to have poor cognitive outcome than children of non- depressed mothers [14]. Poobalan A.S. et al. have demonstrated that treatments for postnatally depressed mothers had some benefits in improving the level of behavioral management problems and cognitive development in children [15]. Hay et al. has also reported a link between postpartum depression and poor child cognitive functioning at both 4 and 11 years [16]. Sinclair and Murray have reported that 5 year old children whose mothers had postpartum depression were found to be more behaviorally disturbed than controls [17]. A recent systematic review has demonstrated that postpartum distress contributes to cognitive and socio-emotional delay in infants from birth to 1 year of age [18]. Murray et al. has also reported an association with postpartum depression and impairments in mother-infant interactions as well as longer term disruption of emotional and cognitive development of the infant [19].

Hadley et al. have found that children of mothers with high symptoms of depression scored significantly lower on the personal-social, fine motor and gross motor scales [20].

The first year of life is a crucial period for a child’s language development. It is one of the most complex developmental challenges and many children do not develop language easily [21]. Several factors play a key role in a child’s acquisition of vocabulary, such as the interactive stimulating environment, socio-economic status and the relationship with the parents [22]. Quevedo et al. have reported that children of mothers who were depressed during the postnatal period were at increased risk of language acquisition problems at 12 months of age [23]. Brennan et al. have reported that the severity and duration of maternal depression increases the behavior problems and vocabulary problems of the children [24]. Maternal age can also influence a child’s language development, as younger mothers may be more patient and talk more to their children whereas older mothers could be more tired and thus talk less to their children [25]. Quevedo et al. have found an association between the parity of the mother and the language ability of children; women with more than two children were more likely to have children with poorer language development [23].

There is paucity of prospective studies regarding the impact of postpartum depression on children in developing countries across the domains of mental development. Our study hypothesis was to assess the detrimental effect of postpartum anxiety and depression on children’s mental development. Therefore, this study aimed to look for evidence of impact of ***postpartum anxiety and depression*** on children’s mental development across all the five domains. Here we have used the term ***postpartum anxiety and depression***, as anxiety is a more prominent feature of PPD than depression that occurs at other times in life [26,27].

## Methods

### Study design, site and duration

This is a quasi-experimental study to assess “Effect of postpartum anxiety and depression on early childhood growth and development,” carried out in two peri-urban, multiethnic, communities of Karachi named Qayoomabad and Manzoor Colony from February 2004 – January 2007. Karachi is the largest city and economic capital of Pakistan with its population representing almost all socio-economic and ethnic groups in Pakistan and is referred to as Mini-Pakistan. The above field sites were chosen with the expectation that the results would probably be more generalizable to other areas of the country.

### Study participants and sampling

All pregnant women who were living in the defined sites were identified by house to house survey. The field workers visited each house and took permission to enter by displaying their identification cards to the residents. Then explained the purpose of the study and inquired if the household had a married woman in the reproductive age group. If there was one she was explained the objective of the study; if she consented to participation her last date of menstrual period was inquired. A total of 651 pregnant women were identified, during February 2004 to December 2005; out of them 420 women were enrolled who had given consent for participation after giving a live childbirth. Detailed information about the recruitment process is given elsewhere [28].

### Instruments used

#### Aga Khan University Anxiety and depression Scale (AKUADS)

This is a screening instrument for anxiety and depression developed from symptoms of anxious and depressed patients noted verbatim in the local lingua franca Urdu and validated in the community keeping the psychiatrists interview as the gold standard [28,29]. It is a 25 item scale (13 psychological and 12 somatic items) that covers most of the clinical features considered characteristic of anxiety and depressive disorders. Each item has four response options (never, sometimes, often, always) scored from 0 to 3. At a cut off score of 19 it has a sensitivity of 74%, specificity of 81%, a positive predictive value of 63% and a negative predictive value of 88% [30]. It has been used in several studies in Pakistan [28-34]. As AKUADS is a screening instrument, confirmation was obtained from a clinical psychologist. Mothers who tested positive on AKUADS (score of 19 or above) and those who were marginally below the cut off score i.e. with scores of 17 and 18 were also interviewed by a clinical psychologist for confirmation of diagnosis according to DSM IV criteria. However, women with a score of 16 and below were considered as not anxious/depressed and were not interviewed by the clinical psychologist.

#### Socio-demographic questionnaire

Mother’s age, religion, ethnicity, education and occupation, husband education and occupation, household monthly income (in Pakistani Rupees), ownership of house, total number of rooms, total number of household members, number of pregnancies, number of live births, number of abortions/stillbirths, number of children who had died, reasons for their death, planned or unplanned current pregnancy, ever used or intention to use of any contraceptive method and autonomy to use contraceptive method- were recorded in the questionnaire.

#### Home environment/Family relationship questionnaire

Noted the presence or absence of stressful home environment, satisfaction with current life, any family/social support to cope with stress, decision maker in the household, mother ever being abused physically or verbally by any family member (including during current pregnancy) and children ever abused physically or verbally by any family member. A variable “domestic abuse” was defined as either mother or children physically or verbally abused by any person in the house.

#### Post-natal questionnaire

Gender of the child, date of birth, weight (in Kg.) of the child, place of birth, person who had conducted the delivery, any complication during child birth, and any complications in the newborn was also filled.

#### Early childhood development (ECD) tool

It comprises of five major components of development of a child i.e. gross motor, fine motor, language, cognitive (perception and learning) and socio-emotional development. This tool is developed by Human Development Program (HDP) at Aga Khan University, designed to monitor the physical and mental growth of children up to age 6 years [35]. Each component is rated as “Delayed, Appropriate or Accelerated”. For the current study last two categories were merged and labeled as “Normal”. Hence each child development index was dichotomized as “Normal versus Delayed”.

### Selection and training of field workers

Women aged 18 years and above residing at the study sites, able to read and write Urdu (lingua franca in Pakistan) and willing to be trained were identified. They were trained in administration of the screening instrument, The Aga Khan University Anxiety and Depression Scale (AKUADS) and other study questionnaires mentioned above. Eleven training sessions on counseling were held over four weeks, each session was of three hours. The trainers included two family practitioners, a psychiatrist and a clinical psychologist. The training encompassed basic information regarding anxiety/depression, stress/anger management and communication/counseling skills. Communication covered principles of active listening, probing and feedback, whereas counseling dealt with supportive, problem-solving and basic cognitive-behavioral techniques. The model used was participatory and facilitatory [36]. Out of the 19 trained women 11 were selected as counselors based on their ability to maintain confidentiality, communicate empathically and permission from family to move freely in the community.

In addition, they were trained in monitoring the growth and development of the indexed baby by measuring weight, height, and in using early childhood development (ECD) tool. Besides this they also provided information to mothers regarding healthy child rearing practices.

### Enrolment and data collection

A field office was established at Qayoomabad. The field workers visited each house in Qayoomabad and adjacent sectors of Manzoor colony which were easily accessible from the field office, to identify pregnant women by inquiring the date of their last menstrual period from women in the reproductive age group. Those found to be pregnant were informed of the objectives of the study and were invited to participate in the study after childbirth and written consent was obtained. This process of identification of pregnant women was carried on- for the first two years of the study. The expected date of delivery was calculated from the date of the last menstrual period. For those women who gave written consent to participate in the study, the field workers started weekly visits when the women reached the estimated 36^th^ week of pregnancy and continued doing so till childbirth. Consent was again obtained from mothers of live births before enrolling them in the study. The socio-demographic, home environment, family relationship and newborn postnatal questionnaires were administered by the field workers within seven to ten days of childbirth. Routine follow-ups were scheduled after 1, 2, 6, 12, 18, 24 and 30 months of childbirth for screening of anxiety/depression among mothers. Child’s health status, physical and mental growth was recorded at every month after birth till the age of 1 year and then quarterly till the end of the study.

Verbal consent was obtained each time before administering AKUADS. Women with AKUADS scores of 17 or above were interviewed by the clinical psychologist for confirmation of anxiety/depression according to DSM IV criteria. Those who were found to be anxious/depressed by the clinical psychologist or who scored 19 or above on AKUADS were offered weekly one-hour counseling sessions for eight weeks. Eight sessions were chosen as one of the earlier studies done by one of our co-author has demonstrated that the effect of counseling peaked at 4 weeks and remained somewhat stable at 8 weeks [37]. The sessions were mostly based on Rogerian principles of presence, active listening, and unconditional positive regard and avoiding sympathy and providing solutions. Very basic cognitive behavioral therapy, supportive and problem-solving counseling was provided [36]. The basics of CBT were included to just introduce them to the relationship between cognition, behavior and feelings. Sessions were conducted at the client’s residence on the day and time of her convenience. Details of counseling sessions are given elsewhere [38].

### Ethics approval

The project was funded by the Aga Khan University Research Council Grant and ethical approval was obtained from the Ethics Committee of the Aga Khan University.

### Data management

Data was double entered using EpiData (version 3.02) package; 10% of the records were randomly checked to assess the quality of data entry. The data entry error rate was observed as less than 3 per 1000 fields entered. The final data was analyzed using the statistical software package SAS 9.2.

### Statistical data analysis

Descriptive statistics were computed for socio-demographic characteristics of parents, home environment and post-natal variables at baseline. Multivariable Generalized Estimating Equations (GEE) logistic regression analysis was conducted to identify parental and child factors independently associated with each early childhood development index [39]. Regression analysis for each early childhood development subscale was started by determining how follow-up month should be modeled in the univariate model; polynomial models were explored for the variable of time- “month of-follow-up ”. For each subscale a multivariable GEE regression model was then built by assessing Maternal A and D, child’s stunted status at each follow-up, and socio-demographic characteristics of parents, home environment and post-natal variables at baseline for inclusion in the univariate GEE regression model. Potential predictor variables with Wald p-value < 0.15 and those of biological significance were selected for inclusion in the multivariable model. In the multivariable model variables that were not significant and not confounding the effect of other variables in the model were removed. QIC and QIC_u_ criteria were also used for model selection, and QIC criteria used for selection of the working correlation structure [40]. Scale examination of continuous predictor variables was done using the quartile analysis technique in the multivariable model [41]. Biologically meaningful interactions of “Maternal Aand D” with the other predictor variables in the model were assessed for significance in the multivariable model. If there was a significant interaction between mother’s depression and follow-up month, impact of mother’s depression at the 2nd month (25th percentile), 6th month (50th percentile) and 12th month (75th percentile) of follow-up respectively was examined. Analysis was conducted using PROC GENMOD on SAS 9.2 [42].

## Results

The study population comprised of 420 pregnant women, with 155 women from Manzoor Colony and 265 from Qayoomabad. Socio-demographic and home environment characteristics of the study population at baseline in both areas are reported in Table 1. Mean (SD) age of women from Manzoor Colony was 27.0 (5.3) years, whereas that of women from Qayoomabad was 25.5 (4.4) years. About 25% of the women from Qayoomabad and 30% from Manzoor Colony were illiterate. Mean (SD) monthly income of husbands in the study population was about 5400 (3233) Rupees ($90). In Manzoor Colony none of the women reported domestic physical abuse, whereas about 1% reported verbal abuse. At Qayoomabad site about 3% women reported physical and 6% verbal abuse. Less than 10% mothers worked for pay or profit (Table 1).

**Table 1:** Socio-demographic and home environment characteristics of parents and children at baseline by study area (N=420)

	**Manzoor colony (N=155)**	**Qayoomabad (N=265)**
**Mean (SD) age of mothers (years)**	27.0 (5.3)	25.5 (4.4)
**Mother’s educational level;** n(%)		
Illiterate	46 (29.7%)	65 (24.5%)
Read & Write Only/Below Matric	51 (32.9%)	116 (43.8%)
Matric or above	58 (37.4%)	84 (31.7%)
**Educational level of husband;** n(%)		
Illiterate	33 (21.3%)	41 (15.5%)
Read & Write Only/Below Matric	47 (30.3%)	92 (34.7%)
Matric or above	75 (48.4%)	132 (49.8%)
**Mean (SD) income of husband (Rupees) (N=417)**^**1**^	5336.1 (3492.2)	5402.7 (3076.4)
**Mean (SD) of total number of children alive**^**2**^**(N=416)**^**3**^	2.2 (2.2)	1.7 (1.6)
**Woman subjected to domestic physical abuse**^**4**^**;** n(%)		
Yes	0 (0%)	8 (3.0%)
No	155 (100%)	257 (97.0%)
**Woman subjected to domestic verbal abuse**^**5**^**;** n(%)		
Yes	2(1.3%)	15 (5.7%)
No	153 (98.7%)	250 (94.3%)
**Mother works for pay or profit;** n(%)		
Yes	13 (8.4%)	18 (6.8%)
No	142 (91.6%)	247 (93.2%)
**Current pregnancy accepted;** n(%)		
Yes	140 (90.3%)	209 (78.9%)
No	15 (9.7%)	56 (21.1%)
**Any complication during delivery;** n(%)		
Yes	44 (28.4%)	96 (36.2%)
No	111 (71.6%)	169 (63.8%)
**Children subjected to domestic physical abuse**^**6**^**;** n(%)		
Yes	0 (0.0%)	17 (6.4%)
No	155 (100.0%)	248 (93.6%)
**Children subjected to domestic verbal abuse**^**7**^**;** n(%)		
Yes	0 (0.0%)	20 (7.5%)
No	155 (100.0%)	245 (92.5%)
**Child’s gender;** n(%)		
Male	74 (47.7%)	145 (54.7%)
Female	81 (52.3%)	120 (45.3%)
**Child had difficulty in breast feeding;** n(%)		
Yes	20 (12.9%)	39 (14.7%)
No	135 (87.1%)	226 (85.3%)

^1^Three women at Qayoomabad site had missing information for husband’s income.

^2^Total number of children alive at the index pregnancy.

^3^Four women at Qayoomabad site had missing information for total number of children alive.

^4^Defined as woman physically abused by any person in the house.

^5^Defined as woman verbally abused by any person in the house.

^6^Defined as children physically abused by any person in the house.

^7^Defined as children verbally abused by any person in the house.

Proportion of mothers depressed at the 1st month of follow-up was 4.8%, at the 2nd month 4.7%, at the 6th month 5.7%, at the 12th month 9.2%, at the 18th month 2.7% and at the 24th month 6.1%. Only 76 mothers could be followed to 30 months and none of these were depressed.

For 29 mothers depression was not recorded at any follow-up, hence these mothers were excluded from further analysis. For six mothers information regarding husband’s income or total number of children alive at the index pregnancy was missing and they were also excluded. Hence complete information was available for 385 (91.6%) mothers for further analysis.

### Child emotional development

Table 2 presents the results of multivariable GEE logistic regression model for child emotional development. Adjusting for other variables in the model, mother’s depression puts the child at about 6-fold risk for being delayed for emotional development (OR_adj_= 5.9, 95% CI: 3.0, 11.9). Similarly when children were stunted they were twice more likely to be delayed for emotional development. (OR_adj_= 2.3, 95% CI: 1.1, 4.8).

**Table 2:** **Multivariable GEE model**^**1 **^**and adjusted odds ratio for child emotional development (N=385)**

**Variables**	**Adjusted odds ratio**	**95%confidence interval (CI)**
**Mother’s depression**		
No	1.0	-
Yes	5.9	(3.0, 11.9)
**Child stunted**		
No	1.0	-
Yes	2.3	(1.1, 4.8)
**Mother’s age**^**2**^	1.5	(1.1, 2.2)
**Mother’s education**		
Literate	1.0	-
Illiterate	1.6	(0.8, 3.1)
**Husband’s education**		
Literate	1.0	-
Illiterate	0.4	(0.2,1.0)
**Husband’s income**		
≥ 3500 Rupees/month	1.0	-
< 3500 Rupees/month	1.8	(0.9, 3.8)
**Total number of children alive**		
≥ 3	1.0	-
<3	1.8	(0.9, 3.8)
**Physical abuse of mother by a person in the house**		
No	1.0	-
Yes	2.5	(0.7, 9.0)
**Any complication during delivery**		
No	1.0	-
Yes	0.5	(0.2, 1.1)

^1^Adjusting for verbal abuse of children by a person in the house, and month-follow-up (modeled as cubic polynomial).

AR(1) correlation structure, QIC =418.08 QIC_u_= 418.92

^2^The reported odds ratio and CI is for every 5 years increase in age.

Our results suggest that higher maternal age adversely affects child’s emotional development. There is evidence that a mother having less than 3 live children at the index pregnancy and a low husband’s income (< 3500 Rupees/months) puts the child at a greater risk for delayed emotional development. There is some evidence that children of illiterate mothers and those of mothers who had suffered from physical abuse were more likely to be delayed for emotional development.

### Child cognitive development

Table 3 presents the results of multivariable GEE logistic regression model for child cognitive development. There was a significant interaction between mother’s depression and follow-up month. At the 2nd month of follow-up there was no significant impact of mother’s depression on cognitive development of children. However, the impact of maternal depression on children’s delayed cognitive development was about 3-folds at the 6th month follow-up (OR_adj_= 3.3, 95% CI: 1.1, 9.9) and was about 7-folds at the 12th month of follow-up (OR_adj_= 6.8, 95% CI: 3.0, 15.7).

**Table 3:** **Multivariable GEE model**^**1 **^**and adjusted odds ratio for child cognitive (perception & learning) development (N=385)**

**Variables**	**Adjusted odds ratio**	**95%confidence interval**
**Mother’s age**		
≥ 30	1.0	-
<30	1.8	(1.1, 3.0)
**Total number of children alive**^**2**^		
<1	1.0	-
≥ 1	1.7	(1.0, 2.7)
**Child sunted**		
No	1.0	-
Yes	1.5	(0.9, 2.3)
**Interaction between mother’s depression & month of follow-up**		
*2nd Month of follow-up*^*3*^		
**Mother depressed**		
No	1.0	-
Yes	1.1	(0.2, 6.2)
*6th Month of follow-up*^*3*^		
**Mother depressed**		
No	1.0	-
Yes	3.3	(1.1, 9.9)
*12th Month of follow-up*^*3*^		
**Mother depressed**		
No	1.0	-
Yes	6.8	(3.0, 15.7)

^1^Adjusting for mother’s & husband’s education respectively and month-follow-up (modeled as cubic polynomial).

Exchangeable correlation structure, QIC =726.82 QIC_u_= 725.82.

^2^Total number of children alive at the index pregnancy.

^3^2nd month of follow-up is 25th percentile, 6th month the median and 12th month the 75th percentile of follow-up month values.

When children were stunted they were at a higher risk for delayed cognitive development (OR_adj_= 1.5, 95% CI: 0.9, 2.3). Children of mothers aged less than 30 years, and those of mothers having one or more live children at the index pregnancy were more likely to be delayed for cognitive development.

### Child language development

Table 4 presents the results of multivariable GEE logistic regression model for child language development. There was a significant interaction between mother’s depression and her husband’s income. Among women who were not depressed there was no significant impact of husband’s low income on child’s language development. Similarly, among women whose husband’s income was ≥ 3500 Rupees/month there was no significant impact of maternal depression on child’s language development. Children of depressed mothers whose husbands income was < 3500 Rupees/month were at more than 5-folds risk of being delayed for language development relative to children whose mothers were not depressed and whose fathers income was ≥ 3500 Rupees/month (OR_adj_= 5.4, 95% CI: 2.3, 12.4).

**Table 4:** **Multivariable GEE model**^**1 **^**and adjusted odds ratio for child language development (N=385)**

**Variables**	**Adjusted odds ratio**	**95%confidence interval**
**Child’s gender**		
Female	1.0	-
Male	1.4	(0.9, 2.2)
**Total number of children alive**^**2**^	1.1	(1.01, 1.3)
**Child stunted**		
No	1.0	-
Yes	1.6	(1.0, 2.4)
**Mother’s education**		
Matric or above	1.0	-
Read & write only/Below matric	1.6	(1.0, 2.7)
Illiterate	1.8	(1.0, 3.3)
**Current pregnancy accepted**		
Yes	1.0	-
No	1.5	(0.9, 2.4)
**Mother works for pay or profit**		
Yes	1.0	-
No	6.9	(1.7, 28.3)
**Study area**		
Manzoor colony	1.0	-
Qayoomabad	3.6	(2.1, 6.3)
**Interaction between mother’s depression & husband’s income**		
Not depressed & income ≥ 3500^3^	1.0	-
Not depressed & income < 3500	1.0	(0.6, 1.6)
Depressed & income ≥ 3500	1.4	(0.6, 3.5)
Depressed & income < 3500	5.4	(2.3, 12.4)

^1^Adjusting for mother’s age and month-follow-up (modeled as linear polynomial).

AR(1) correlation structure, QIC =861.41 QIC_u_= 858.10.

^2^Total number of children alive at the index pregnancy; this variable is continuous (linear) in this model.

^3^Rupees/month.

When a child was stunted he/she was more likely to be delayed for language development (OR_adj_= 1.6, 95% CI: 1.0, 2.4). There is evidence that male children were at higher risk for delayed language development relative to female children. Children of illiterate women were 1.8 times, and those of with education below high school (matric) were 1.6 times more at risk of being delayed for language development. The risk of children’s delayed language development increased as the number of mother’s living children increased.

There is a very strong evidence that children of mothers who did not work for profit and some marginal evidence that children of mothers who did not accept the current pregnancy were at a higher risk for delayed language development.

Adjusting for other variables in the model children of mothers living in Qayoomabad were at more than 3-folds risk for delayed language development relative to those from Manzoor Colony.

### Child gross motor development

Table 5 presents the results of multivariable GEE logistic regression model for child gross motor development. There was again a significant interaction between mother’s depression and the month of follow-up. At the 2nd and the 6th month of follow-up there was no significant impact of maternal depression on gross motor development of children. However, the impact of maternal depression on children’s delayed gross motor development was about 3-folds at the 12th month of follow-up (OR_adj_= 2.8, 95% CI: 1.2, 6.6).

**Table 5:** **Multivariable GEE model**^**1 **^**and adjusted odds ratio for child gross motor development (N=385)**

**Variables**	**Adjusted odds ratio**	**95%confidence interval**
**Child’s gender**		
Female	1.0	-
Male	1.7	(1.05, 2.9)
**Child stunted**		
No	1.0	-
Yes	1.7	(0.9, 3.0)
**Child had difficulty in breast feeding**		
No	1.0	-
Yes	1.7	(0.8, 3.7)
**Mother’s education**		
Matric or above	1.0	-
Below Matric^2^	1.8	(1.01, 3.2)
**Study area**		
Manzoor Colony	1.0	-
Qayoomabad	1.6	(0.8, 2.9)
**Interaction between mother’s depression & month of follow-up**		
*2nd Month of follow-up*^*3*^		
**Mother depressed**		
No	1.0	-
Yes	1.5	(0.3, 7.0)
*6th Month of follow-up*^*3*^		
**Mother depressed**		
No	1.0	-
Yes	0.7	(0.1, 3.4)
*12th Month of follow-up*^*3*^		
**Mother depressed**		
No	1.0	-
Yes	2.8	(1.2, 6.6)

^1^Adjusting for mother’s age, total number of children alive, domestic abuse and month-follow-up (modeled as cubic polynomial).

Exchangeable correlation structure, QIC =605.61 QIC_u_= 602.36.

^2^Below Matric refers to any level of education below Matric, Read & Write Only or Illiterate.

^3^2nd month of follow-up is 25th percentile, 6th month the median and 12th month the 75th percentile of follow-up month values.

When children were stunted they were more likely to be delayed for gross motor development (OR_adj_= 1.7, 95% CI: 0.9, 3.0). Male children were at higher risk for delayed gross motor development relative to female children. There is some evidence that children who have had difficulty in breast feeding were at a higher risk for delayed gross motor development. Children of illiterate mothers or those whose mothers had education less than matric were 1.8 times more likely to be delayed for gross motor development.

### Child fine motor development

Table 6 presents the results of multivariable GEE logistic regression model for child fine motor development. There was a significant interaction between maternal depression and the month of follow-up. At the 2nd and the 6th month of follow-up there was no significant impact of maternal depression on children’s fine motor development. At the 12th month there is evidence of an adverse effect of maternal depression on children’s fine motor development; however this effect is not significant at 5% level. At the 18th month of follow-up there is a significant impact of maternal depression on children’s delayed fine motor development; children of depressed mothers being at a 4-folds risk (OR_adj_= 4.0, 95% CI: 1.4, 11.3).

**Table 6:** **Multivariable GEE model**^**1 **^**and adjusted odds ratio for child fine motor development (N=385)**

**Variables**	**Adjusted odds ratio**	**95%confidence interval**
**Child stunted**		
No	1.0	-
Yes	1.8	(1.03, 3.0)
**Child had difficulty in breast feeding**		
No	1.0	-
Yes	1.8	(1.01, 3.4)
**Mother’s education**		
Matric or above	1.0	-
Below matric^2^	1.4	(0.8, 2.4)
**Mother’s age**^**3**^	1.2	(0.9, 1.6)
**Total number of children alive**^**4**^		
≥ 3	1.0	-
<3	1.7	(0.95, 3.0)
**Any complication during delivery**		
No	1.0	-
Yes	0.6	(0.3, 1.04)
**Physical abuse of mother by a person in the house**		
No	1.0	-
Yes	2.4	(0.9, 6.7)
**Husband’s income**		
≥ 3500 Rupees/month	1.0	-
< 3500 Rupees/month	2.2	(1.4, 3.5)
**Interaction between mother’s depression & month of follow-up**		
*2nd Month of follow-up*^*5*^		
**Mother depressed**		
No	1.0	-
Yes	0.4	(0.1, 1.6)
*6th Month of follow-up*^*5*^		
**Mother depressed**		
No	1.0	-
Yes	0.7	(0.2, 2.2)
*12th Month of follow-up*^*5*^		
**Mother depressed**		
No	1.0	-
Yes	1.6	(0.6, 4.2)
*18th Month of follow-up*^*6*^		
**Mother depressed**		
No	1.0	-
Yes	4.0	(1.4, 11.3)

^1^Adjusting for husband’s education, physical abuse of children by a person in the house and month-follow-up (modeled as linear polynomial).

Exchangeable correlation structure, QIC =623.48 QIC_u_= 626.56.

^2^Below Matric refers to any level of education below Matric, Read & Write Only or Illiterate.

^3^The reported odds ratio and CI is for every 5 years increase in age.

^4^Total number of children alive at the index pregnancy.

^5^2nd month of follow-up is 25th percentile, 6th month the median and 12th month the 75th percentile of follow-up month values.

^6^Impact of Maternal A & D was assessed at 18 months as the effect was not significant at 2 and 6 months, and only an evidence of an impact was seen at 12 months. The later indicate that a significant effect of maternal A & D will manifest itself as the follow-up time increases.

When children were stunted they were at a higher risk for delayed fine motor development. (OR_adj_= 1.8, 95% CI: 1.0, 3.0). Difficulty in breast feeding was significantly associated with higher risk for delayed fine motor development. There is some evidence that maternal illiteracy or education less than matric and increasing maternal age is a risk factor for children’s delayed fine motor development. Children of mothers having less than 3 live children at the index pregnancy were more likely to be delayed for fine motor development. There is evidence that physical abuse of mothers puts the children at more than twice risk for delayed fine motor development. Our results provide some evidence for complications during delivery being protective for fine motor development.

## Discussion and conclusions

In this study we have found that, if the mother was depressed the child was six times more at risk of being delayed for ***emotional development*** relative to when the mother was not depressed. This could be due to the care provided by a mother to her child have been compromised by having less interaction with their children if she is depressed.

We also found that stunting puts children twice at risk of being delayed for emotional development. There was evidence that low family income and maternal illiteracy were associated with delayed emotional development of children. In addition, children of mothers who had suffered from physical abuse by a person in the house were more likely to be delayed for emotional development. These can be explained if the mother is pre-occupied about financial constraint or difficulties with her partner or family, it can be hard to focus on the child’s experience.

Several studies focusing on social behavior as the indicator of infant socio-emotional development have found an association with maternal postpartum depression and impairment of emotional development of the infant [18,19,43-46]. The reason could be depressed mothers remains preoccupied with their own feelings, causing them to miss infant cues and appear withdrawn and disengaged. Grace et al. have also reported that postpartum depression can cause disruptive behavior in the first year of life [47]. Nutritional stunting, which is rampant in many low-income developing countries, is known to be associated with both emotional-social and cognitive development among young children and to be a strong predictor of subsequent cognitive development and school performance [48].

Our study found that children of mothers who were depressed during the postpartum period and whose husband’s income was less than 3500 Rupees/month were more than 5 times at risk of being delayed for ***language development***. This could be depressed mothers are more worried about the financial constraint and are less vocal and have less interaction with their children. In our study there was evidence that male children were at higher risk for delayed language development relative to female children. The possible explanation could be depressed mothers treat their sons and daughters differently. However, this gender effect needs to be tested in future studies. Our study also indicates that low maternal education adversely effects child’s language development. The risk of children’s delayed language development increased as the number of mother’s living children (at the index pregnancy) increased. This could be because a mother with other children will have less time to spend with the indexed child.

Quevedo et al. have found that children of mothers who were depressed were at higher risk of language acquisition at 12 months of age [23]. Brennan et al. have reported that the severity and duration of maternal depression increases the behavior problems and vocabulary problems of the children [21]. Older women and women with more than two children were more likely to have children with poorer language development [23]. Maternal age could influence a child’s language directly as younger mothers are more interactive with their children [20]. Reilly S et al. have found that language development was predicted by low socio-economic status, multiple births (e.g. twins had poorer scores), gender (e.g. girls better cognitive scores than boys) and family history of language difficulties [24].

In our study we found a significant adverse impact of mothers’ depression on ***cognitive development*** of children at 6 months; this impact almost doubled at 12 months. This could be associated with the quality and extent of maternal interaction/communication with the child, as it is compromised with depressed mothers. In addition, our results indicate that children when stunted were at a higher risk of being delayed for cognitive development. We also found that children of mothers aged less than 30 years were more likely to be delayed for cognitive development relative to children of mothers aged 30 years or older.

Murray et al. have reported that postpartum depression, especially if chronic, poses a risk for poor cognitive development in the child [19]. In India, Patel et al. found that infants whose mothers had postpartum depression had significantly lower mental scores- than infants of non-depressed mothers [49]. Similarly, a study of mothers in Barbados found that infants of mothers with postpartum depression at 7 weeks had lower social and cognitive performance at 6 months [50]. McLearn et al. have also reported that infants of mothers with anxiety-mood disorders have relatively poorer cognitive, social-emotional outcomes and relatively more behavioral problems than children of normal mothers [51]. Very recently a systematic review conducted by Kingston et al. has also reported that postpartum distress contributes to cognitive and socio-emotional delay in infants from birth to 1 year of age [18]. Murray L has reported an adverse effect on global cognitive functioning by low maternal education [52].

We found a significant adverse impact of maternal depression on ***gross motor development*** of children at 12 months. However, there was no significant impact of maternal depression on children’s gross motor development at 2nd and 6th months respectively. This could be depressed mothers provide less optimal levels of stimulation for their infants and fewer opportunities to physically explore their surroundings. Moreover, caregiving demands at this stage may be particularly difficult for mothers who are already burdened by depressive symptoms. The other associated factors mentioned earlier like low level of mother’s education and child being stunted had a detrimental effect on gross motor development. We also found that male children were at higher risk for delayed gross motor development relative to female children.

Regarding children’s ***fine motor development*** some marginal association between maternal depression and delayed development was observed at the 12^th^ month follow-up. At the 18^th^ month follow-up there was a significant impact of maternal depression on fine motor development; children of depressed mothers being at a 4-folds risk. The possible explanation could be by 12 months the impact of environmental influences is often strong enough to create individual differences in development. In our study there was evidence that physical abuse of mothers by a person in the house puts the children at more than twice risk for delayed fine motor development. Our results also indicated that increasing maternal age, low level of maternal education, husband’s low income, and child being stunted had adverse effects on fine motor development.

Hadley C et al. have reported that children of mothers with high symptoms of depression scored significantly lower on the personal-social, fine motor and gross motor scales, and consequently on the overall developmental score [20]. They have also reported that stunted children scored lower on all sub-scales but this was significant only for the gross motor scale. In addition, they reported that children living in households with above the median household goods- scored relatively better on the personal-social scale and the gross motor scale. Their study further indicates that even after controlling for the impact of nutritional stunting, maternal depression remains a significant predictor of children’s developmental outcomes among infants 3–24 months [25].

Similarly, our study has also found that maternal depression is associated with adverse outcomes of their children’s mental development on all sub-scales. This effect becomes more prominent in combination with other known associated factor for depression like low family income.

### Strengths

This study had number of strengths. Firstly it is community-based. Secondly it was a prospective longitudinal study with reasonable sample size with repeated assessments of maternal depression over the course of follow-up with serial assessments of all parameters of infant and child development. Thirdly, community workers were the counselors. Fourth, the instrument which we used for anxiety/depression i.e. AKUADS which is a scale locally developed and validated in the community and covers most of the clinical features considered characteristic of anxiety and depressive disorders in general. Its content validity has been compared with WHO’s SRQ and has been published [53].

### Limitations

One of the limitations of our study was that those women who were enrolled towards the end of the study could not be followed for a longer period of time. Secondly we have a large lost to follow-up rate due to frequent change in residence and refusals after having initially consented due to stigma associated with mental illness. Another limitation was that, this study was conducted in two underprivileged urban communities; hence, the study participants may not completely represent the city population. Lastly the child’s development was mostly reported by the mother as the child was not under observation all the time, though an effort was made by the community counselor to confirm it on her visit.

### Implications and recommendations

The object relation theories all espouse that the development of the Psychic structure of an infant is dependent on the relationship he has with the mother or any other primary care giver. It is also believed that the relationship during this time provides a template for the growing individual for all future relationships. This template is complete by the age of 3 years; all future events and experiences sculpt this template, but the basic structure remains the same, though it can be modified by certain relationships in later life.

The relationship a mother develops with the infant/child is dependent on the mothers own emotional health. Our study agrees with this belief. Projecting it on a larger scale it can be inferred that if we want to promote development of mentally healthy individuals, we need to intervene at this crucial time of early child development. It is suggested that in prenatal and postnatal visits the health care providers should detect and treat mothers who suffer from emotional disorders as is done with other factors that make a mother or her infant vulnerable.

### What this study adds

Additive value of the current study is that we have used the instrument AKUADS for screening anxiety/depression of mothers which is locally developed and validated in the community and confirmation was obtained from a clinical psychologist. Moreover, it has a high linguistic validity in our population, community women can be easily trained in its use and it has been used in several studies in Pakistan. Secondly, the tool ECD is also locally developed tool, designed to monitor growth and development of our local children. Therefore, mostly the results could be a reflection of true picture for local context. Majority of existing studies have assessed some components of mental development. However, in our study we have assessed all the five domains of mental development. In addition, the procedure for the multivariable analysis used in this study is a sophisticated statistical method that takes the longitudinal (follow-up) structure of the data into account, and provides an adequate analysis of the data.

## Competing interests

The authors declared that they have no competing interests.

## Authors’ contributions

NSA conceived and designed the study and prepared the manuscript. BSA has made substantial contributions to conception and design of the study and provided intellectual feedback throughout and has reviewed the manuscript critically for intellectual content. SM has analyzed the data, interpreted the results, has written the results section and contributed in review of the manuscript. AK has worked as senior project coordinator and contributed in acquisition of data and its management. All authors read and approved the final manuscript.

## Pre-publication history

The pre-publication history for this paper can be accessed here:

http://www.biomedcentral.com/1471-244X/13/274/prepub
